# Purification and Characterization of α-Amylase from *Bacillus simplex* BCHCNZ282B

**DOI:** 10.3390/cimb48070699

**Published:** 2026-07-10

**Authors:** Nazenin Karakoç, Sema Agüloğlu Fincan, Barış Enez, Veysi Ortakaya

**Affiliations:** 1 Department of Molecular Biology and Genetics, Faculty of Sciences, Dicle University, Diyarbakir 21070, Türkiye; naze6232@gmail.com (N.K.); semaagul@dicle.edu.tr (S.A.F.);; 2 Veterinary Health Department, Vocational School of Food, Agriculture and Livestock, Bingol University, Bingol 12000, Türkiye

**Keywords:** *B. simplex*, α-amylase, purification, enzyme characterization

## Abstract

Microbial α-amylases are widely used in industrial biotechnology due to their catalytic efficiency, stability, and cost-effective production. Identifying sources of newly isolated bacterial strains with desirable biochemical properties remains important for improving industrial enzyme applications. In this study, a newly isolated strain, *Bacillus simplex* BCHCNZ282B, isolated from soil samples collected in Ergani (Diyarbakır, Turkey), was investigated for its extracellular α-amylase production. The enzyme production conditions were optimized, followed by purification involving ammonium sulfate precipitation, dialysis, and final purification by starch-affinity chromatography. The purified enzyme was biochemically characterized in terms of optimal activity conditions; stability; kinetic parameters; and the effects of metal ions, inhibitors, and detergents. Optimal α-amylase production was obtained at 35 °C and pH 7.0 after 36 h of incubation. The optimum activity of the purified enzyme was attained at 50 °C and pH 7.0. Sequential purification resulted in a six-fold increase in specific activity and a final recovery yield of 19%. The enzyme’s molecular mass was 70 kDa. Increased enzyme activity was obtained in the presence of Mg^2+^ and Mn^2+^, while EDTA was inhibitory to enzyme activity. The purified enzyme maintained high relative activity from pHs 7.0 to 9.0 and was active at all temperatures studied. In addition, enzyme activity was observed even in the presence of some of the tested detergents. The α-amylase produced by *B. simplex* BCHCNZ282B possessed many biochemical properties, such as stability in a wide range of pH values, high-temperature activity, and resistance to several detergents, which indicate many potential biotechnological applications of the enzyme. Nevertheless, further research on genomic characterization and structural analysis should be conducted to establish the industrial application of the enzyme.

## 1. Introduction

Recent advancements in biotechnology have significantly increased the use of enzymes in industrial applications [1]. Enzyme-based industrial processes are generally safer, cleaner, more environmentally friendly, and economically advantageous compared with conventional methods [2]. Enzymes also exhibit higher catalytic efficiency and specificity than chemical catalysts [3,4,5]. An enzyme must be non-allergenic, non-toxic, cost-effective to produce, and applicable across a wide range of sectors to be suitable for industrial use [5]. Enzymes are obtained from plants, animals, and microorganisms [6]. Among these, microbial enzymes are most commonly preferred. Most enzymes used in industry are of microbial origin. Compared with plant- and animal-derived enzymes, microbial enzymes are more cost-effective, exhibit greater biochemical diversity, are easier to genetically manipulate, can be immobilized on water-insoluble substrates, and are more stable under biotechnological conditions. These advantages make microorganisms the preferred source for industrial enzyme production [7,8,9].

Following proteases, amylases constitute the enzyme group with the second-largest market share within the global enzyme industry, accounting for about 30% of the market. They are considered some of the most significant enzymes in industrial biotechnology [10,11]. The alpha-amylase enzyme (EC number: 3.2.1.1) acts as a glycoside hydrolase that catalyzes hydrolysis of internal glycosidic bonds formed via α-1,4 linkage found in compounds such as starch, glycogen, amylose, and amylopectin. During the reaction, polysaccharides are broken down into simpler carbohydrates such as maltose, maltotriose, and dextrin. These products can then be further broken down to glucose using other amylolytic enzymes. Efficient breakdown of starch to fermentable sugar using the α-amylase enzyme explains its widespread use in various sectors, such as food, beverage, textile, paper, detergents, and bioethanol production industries [12].

Amylases play a vital role in various industrial uses. Their primary fields of use include the food, pharmaceutical, paper, textile, detergent, and bioenergy industries [13,14]. Currently, α-amylases are widely utilized in key biotechnological industries, such as paper, food, textile, and detergent manufacturing, as well as in silage production, alcohol fermentation, starch hydrolysis, clinical diagnostics, pharmaceuticals, and analytical chemistry [10,15]. The main advantage of using microorganisms for amylase production lies in the potential for economical large-scale production and the ease of manipulating microbes to obtain enzymes with desired properties. Numerous microbial amylases are commercially available, and they have almost totally replaced chemical hydrolysis in the starch-processing industry [16].

While there are plenty of well-studied *Bacillus* species concerning their abilities to produce industrial α-amylases, little is known about the ability to produce α-amylases and the enzymatic properties of *B. simplex*. Moreover, although many articles have addressed enzyme production optimization, few studies have included enzyme purification and comprehensive biochemical characterization. Thus, further research of novel *B. simplex* strains can lead to the identification of promising enzymes with useful catalytic and stability properties.

In this paper, the *Bacillus simplex* BCHCNZ282B, isolated from soil samples taken from Makam Mountain (Ergani, Diyarbakır, Türkiye), was studied for its ability to produce extracellular α-amylase. Production conditions were optimized, followed by enzyme purification using starch-affinity chromatography and biochemical characterization. This study particularly focused on activity determination, enzyme stability, kinetic parameters, and the effects of metal ions, inhibitors, and detergents on the enzyme.

## 2. Materials and Methods

### 2.1. Bacterial Isolation and Cultivation

Nutrient Agar (NA; Merck, Darmstadt, Germany) medium was used to isolate bacterial strains belonging to the genus *Bacillus*. Soil samples were collected from various points on Makam Mountain, located in the Ergani district of Diyarbakır province. Surface debris was removed, and approximately 10 g of soil was taken from a depth of 2–5 cm using a sterile spatula, placed into sterile glass jars, and stored at +4 °C until use. One gram of each soil sample was transferred into test tubes with 9 mL of sterilized saline solution. The soil samples were vortexed vigorously for 5 min to obtain a homogeneous suspension of the soil. Incubation was carried out at 37 °C for 60 min to allow microorganisms’ dispersion into the aqueous medium. Serial dilutions were made from 10^−1^ to 10^−9^, and 0.1 mL of the dilution from 10^−3^ to 10^−9^ was plated on Nutrient Agar plates. The plate that had isolated colonies was chosen for purification. The plates were incubated at 37 °C for 5 days. Colonies with different morphologies were subcultured on NA medium to obtain pure cultures.

### 2.2. Bacterial Identification

Morphological, physiological, and biochemical tests were initially conducted to characterize the bacteria. The Gram-positive, rod-shaped, motile, hemolytic, and catalase-positive strain with α-amylase enzyme activity was identified by sequencing the bacterial 16S rRNA gene at Ref-Gen Biotechnology Services (METU Technopolis, Ankara, Türkiye), a commercial company providing genetic and sequencing services. In brief, DNA was extracted from the bacterial strain and amplified with universal bacterial primers for the 16S rRNA gene. Then, the PCR amplicons were sequenced. The sequences were aligned with the sequences available in the NCBI database using the BLAST 2.17.0. algorithm. The BLAST analysis was performed by Ref-Gen Biotechnology Services (METU Technopolis, Ankara, Türkiye).

### 2.3. Enzyme Production

For enzyme activity assays, a bacterial culture grown in nutrient broth (NB; Merck, Darmstadt, Germany) prepared with tap water was incubated for 36 h and centrifuged at 10,000 rpm for 10 min using a Sigma 2K15 refrigerated centrifuge (Sigma Laborzentrifugen GmbH, Osterode am Harz, Germany). After centrifugation, the cell-free culture supernatant was collected and used as the crude extracellular α-amylase preparation for all subsequent analyses. The absorbance at 460 nm was used to measure bacterial absorbance during enzyme production, while α-amylase activity was measured using the DNS method at an absorbance of 489 nm.

#### 2.3.1. Effects of Incubation Time, Temperature, and pH on α-Amylase Production

Cultures were preincubated with media at selected pH values, without changing other cultivation conditions, to test the impact of pH on enzyme synthesis. Cultures were grown at various temperatures while keeping other conditions constant to optimize temperature conditions. The enzyme synthesis in the cultures grown under standard cultivation conditions served as the control for comparison. The *B. simplex* BCHCNZ282B strain was cultivated in nutrient broth (NB) medium at pH 7.0 for 4–72 h to elucidate the effect of the incubation time on α-amylase production. Samples were collected at predetermined time intervals (every 4 h), and enzymatic activity along with total protein concentration were determined. The obtained data were used to calculate the specific activity (U/mg). The microorganism was grown in NB medium maintained at a constant pH of 7.0 at different temperatures ranging from 25 to 55 °C to evaluate the effect of temperature on enzyme synthesis. α-amylase activity and protein content were measured in the culture samples obtained at each temperature, and the corresponding calculations were performed. The bacterial growth took place in nutrient broth (NB) at an initial pH level between 3.0 and 10.0 for studying the influence of the medium pH on α-amylase synthesis. The initial pH of the medium was set prior to sterilization by adding either HCl or Tris base to achieve the necessary level of pH. Afterwards, the media were sterilized and incubated under similar cultivation conditions. There were no signs of precipitation during the medium preparation process.

#### 2.3.2. Effect of Different Carbon Sources on α-Amylase Production

The effect of various carbon sources on α-amylase activity was investigated by preparing 25 mL of nutrient broth (NB) medium in 100 mL Erlenmeyer flasks, supplemented with 1% (*w*/*v*) of commercially obtained high-purity carbon sources, including starch, glucose, sucrose (Merck, Darmstadt, Germany), lactose, maltose, and fructose (Sigma-Aldrich, St. Louis, MO, USA). The control culture consisted of standard nutrient broth (NB) medium without modification of the original formulation. The media were sterilized via autoclaving. Each flask was inoculated with 2 mL of bacterial culture and incubated in a shaking water bath at 37 °C for 36 h. After incubation, the samples were collected, and the absorbance values were measured at 460 nm using a spectrophotometer (UV-6450; Jenway, Stone, UK) to determine enzyme activity.

#### 2.3.3. Effect of Different Nitrogen Sources on α-Amylase Production

The effect of various nitrogen sources on α-amylase activity was evaluated by preparing 25 mL of NB medium in 100 mL Erlenmeyer flasks, supplemented with high-purity nitrogen sources at a 1% (*w*/*v*) ratio, including tryptone (Sigma-Aldrich, St. Louis, MO, USA), ammonium nitrate (Merck, Darmstadt, Germany), ammonium chloride (Merck, Darmstadt, Germany), peptone (Oxoid, Basingstoke, UK), and ammonium sulfate (Merck, Darmstadt, Germany). In all optimization experiments, standard nutrient broth (NB) medium was used as the control condition. The media were sterilized via autoclaving. Each flask was inoculated with 2 mL of bacterial culture and incubated in a shaking water bath at 37 °C for 36 h. After incubation, the absorbance values were recorded at 460 nm using a spectrophotometer.

#### 2.3.4. Effect of Growth Media on α-Amylase Production

Caso Bouillon, LB Miller (Sigma-Aldrich, St. Louis, MO, USA), and Tryptone Wasser broths were individually prepared and used as culture media to evaluate the effects of different medium compositions on enzyme production. All optimization experiments were conducted using the same standard nutrient broth (NB) medium as the control condition.

### 2.4. α-Amylase Activity Assay

The α-amylase activity was determined using the Bernfeld method [17]. A reaction mixture consisting of 100 μL of enzyme supernatant and 200 μL of 0.5% starch solution prepared in 0.1 M Tris–HCl buffer (Sigma-Aldrich, St. Louis, MO, USA) (pH 7.0) was incubated at 37 °C for 30 min. After incubation, 400 μL of DNS reagent (3,5-dinitrosalicylic acid (Sigma-Aldrich, St. Louis, MO, USA) was added to stop the reaction, followed by boiling for 5 min. The reaction mixture was then diluted with 3 mL of distilled water, and the absorbance was measured at 489 nm using a spectrophotometer. Reducing sugar concentrations were determined from a calibration curve prepared using known concentrations of maltose. The absorbance values obtained from the DNS assay were converted into maltose equivalents using the corresponding standard curve. One unit (U) of α-amylase activity was defined as the amount of enzyme required to release 1 µmol of reducing sugar (expressed as maltose equivalents) per minute under the assay conditions, as determined using the DNS method. The specific activity was calculated by dividing the enzyme activity (U) by the protein content (mg) and expressed as U mg^−1^ protein.

### 2.5. Protein Concentration Determination

The protein concentration in enzyme samples was determined using the Lowry method [18]. Each tube was filled with 5 mL of an alkaline reagent, and then 50 μL of the enzyme and 450 μL of distilled water were added. After incubation in the dark for 30 min, the absorbance was measured at 660 nm.

### 2.6. Purification of α-Amylase

*Bacillus simplex* BCHCNZ282B was fermented to produce the enzyme using the optimized parameters, as established in this experiment. The culture broth after cultivation was centrifuged at 10,000 rpm for 10 min to remove the bacterial cells. The cell-free culture supernatant, containing the extracellular α-amylase, was used as the raw enzyme extract. As α-amylase was produced in the culture medium, the cell disruption step was unnecessary. The extracellular crude form of α-amylase in the cell-free culture supernatant served as the source for the purification process. Bacterial α-amylase was purified using a mixture of ammonium sulfate precipitation, dialysis, and starch-affinity chromatography.

#### 2.6.1. Ammonium Sulfate Precipitation

Ammonium sulfate [(NH_4_)_2_SO_4_] was gradually added to the crude enzyme extract to achieve saturation levels of 40%, 50%, 60%, 70%, and 80% under continuous stirring in an ice bath (at approximately 4 °C).

#### 2.6.2. Dialysis

The precipitated enzyme solution was placed in a 12 kDa molecular mass cut-off (MWCO) dialysis tubing membrane and stirred overnight at 4 °C against distilled water using a magnetic stirrer. The resulting dialysate was then used for protein determination and enzyme activity assays.

#### 2.6.3. Starch-Affinity Chromatography

After the precipitation of ammonium sulfate (70%) and dialysis, 50 mL of the dialyzed enzyme extract was purified through starch-affinity chromatography. Briefly, 2 g of corn starch was mixed with the dialyzed enzyme extract and shaken at 4 °C at 140 rpm for 1 h to allow the binding of the α-amylase with the starch. Following that, the mixture was centrifuged at 10,000 rpm for 10 min at 4 °C using Sigma 2K15 refrigerated centrifuge (Sigma Laborzentrifugen GmbH, Osterode am Harz, Germany). The supernatant was discarded, and the starch was washed with 50 mL of precooled 0.1 M Tris–HCl buffer (pH 7.0) to remove unbound protein and again stirred at 140 rpm for 5 min at 4 °C. Subsequently, the mixture was centrifuged under the same condition. The bound α-amylase was eluted by resuspending the starch pellet in 50 mL of prewarmed 0.1 M Tris–HCl buffer (pH 7.0) and shaking the mixture at 40 °C at 140 rpm for 1 h. Following the last step of centrifugation at 10,000 rpm for 10 min at 4 °C, the supernatant comprising the pure enzyme was harvested and employed in further characterization assays or stored at 4 °C for later use [19].

### 2.7. SDS-PAGE Analysis and Determination of Molecular Mass of α-Amylase of B. simplex BCHCNZ282B

The molecular mass of purified α-amylase was assessed via sodium dodecyl sulfate–polyacrylamide gel electrophoresis (SDS-PAGE). The enzyme fraction from starch-affinity chromatography was run through an electrophoresis system utilizing 5% stacking gel and 10% separating gel. The calibration curve of protein molecular mass based on relative mobility (Rf) values was constructed using the molecular mass standards, and then it was used to determine the molecular mass of purified α-amylase.

In order to verify α-amylase activity, the separate starch-containing SDS-PAGE was carried out with the inclusion of 3% (*w*/*v*) soluble starch in the separating gel. Dialyzed enzyme fraction, starch-affinity chromatography fraction and commercial α-amylase (positive control) were added with sample loading buffer, run on the gel, and then electrophoresis was performed. After electrophoresis, the starch-containing gel was stained with Lugol’s iodine solution for 30 min. Clear bands against dark-blue background were considered to be the sites of starch degradation.

### 2.8. Effects of Various Inhibitors on the Activity of Purified α-Amylase

Test tubes containing 50 µL of the enzyme and selected inhibitor compounds were incubated at 37 °C for 30 min to evaluate the effects of specific inhibitors on purified α-amylase activity. Subsequently, starch was added, and the enzyme activity was measured. The inhibitors PMSF, methanol, 2-mercaptoethanol, EDTA, and ethanol were tested at final concentrations of 1, 5, and 10 mM. A control assay containing no inhibitor was used as 100% activity. Residual enzyme activities were expressed as percentages relative to the control activity.

### 2.9. Effects of Various Metal Ions on Activity of Purified α-Amylase

An amount of 50 µL of purified α-amylase was mixed with 5 µL of 50 mM stock solutions of ZnCl_2_, HgCl_2_, CuCl_2_, MnCl_2_, CaCl_2_, and MgCl_2_ (MerckGermany) to determine the effects of metal ions on enzyme activity. The mixtures were pre-incubated for 30 min, starch was added, and α-amylase activity was measured.

### 2.10. Effects of Detergents on Activity of Purified α-Amylase

Tween-40, Tween-60, Tween-80, Triton X-100, SDS, Brij (Sigma-Aldrich, St. Louis, MO, USA), and a commercially available enzyme-free laundry detergent (Omomatik, İstanbul, Türkiye) were added to the reaction mixtures at a final concentration of 0.5% (*v*/*v*) to assess the effects of different detergents on purified α-amylase activity.

### 2.11. Determination of pH Stability of Purified α-Amylase

The pH stability of purified α-amylase was evaluated using different buffer systems: 0.1 M citric acid–Tris buffer (pH 4.0 and 5.0), 0.1 M Tris–HCl buffer (pH 6.0, 7.0, and 8.0), and 0.1 M buffer (pH 9.0). The purified enzyme was preincubated with each buffer for 60 min. Following preincubation, the substrate was added, and the residual α-amylase activity was determined under standard assay conditions.

### 2.12. Determination of Thermal Stability of Purified α-Amylase

The purified α-amylase enzyme was pre-incubated at 40 °C, 50 °C, and 60 °C for various periods of time ranging from 0 to 120 min to assess the stability of the enzyme against heat. During each time point of sampling, an aliquot was collected, and the residual activity of the enzyme was assessed according to standard procedure based on the Bernfeld method. The activity recorded before pre-incubation (0 min) was considered 100% and other activities were recorded in percentages of that value.

## 3. Results and Discussion

### 3.1. Bacterial Identification

**Figure 1:**
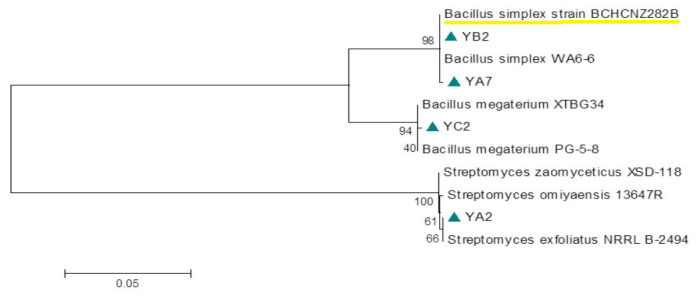
Phylogenetic tree based on 16S rRNA gene sequence analysis showing the taxonomic position of *Bacillus simplex* BCHCNZ282B relative to closely related *Bacillus* species.

Identifying enzyme-producing microorganisms is crucial to study their capabilities from a biotechnological perspective and compare them with other known strains. As such, molecular identification of the strain was carried out using sequencing of the 16S rRNA gene. The isolated bacterium was identified via 16S rRNA analysis at Ref-Gen (METU Technocity/Ankara). The phylogenetic tree of *B. simplex* BCHCNZ282B is depicted in Figure 1. The sequence analysis identified the strain as belonging to the genus *Bacillus*.

### 3.2. Effects of Different Incubation Times, Temperatures, and pH Values on α-Amylase Production

**Figure 2:**
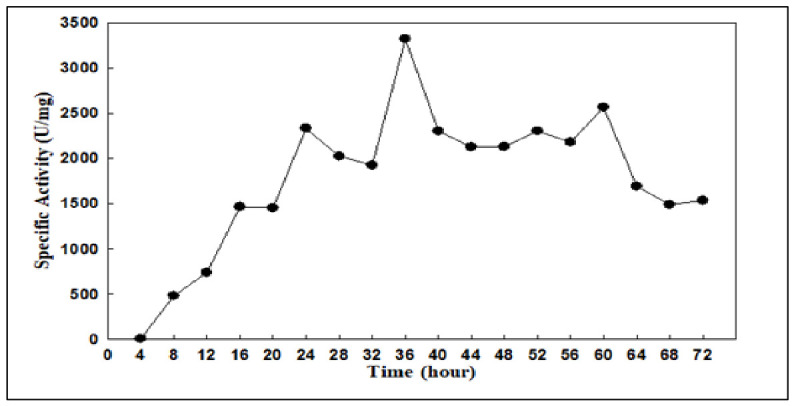
Effect of incubation time (4–72 h) on α-amylase production by *Bacillus simplex* BCHCNZ282B cultivated under optimized culture conditions. Enzyme activity is expressed as specific activity (U mg^−1^).

It is crucial to optimize the cultivation time to maximize the enzyme yield, as enzyme production occurs on a growth-phase basis. Thus, the production of α-amylase by *B. simplex* BCHCNZ282B was observed during a cultivation time of 72 h. Figure 2 shows that α-amylase production occurred between 4 and 72 h, with the highest specific activity observed at 36 h, followed by a gradual decline thereafter. This could be due to nutrient exhaustion, buildup of metabolic waste products, or proteolysis of extracellular proteins in the stationary phase. Agüloğlu Fincan et al. [20] reported that maximum α-amylase production by the thermophilic *Bacillus licheniformis* SO-B3, isolated from hot spring mud in Şırnak, occurred at 36 h. Similarly, Ray et al. [21] observed the highest α-amylase production by *Bacillus* brevis MTCC 7521, isolated from brick kiln soil, at 36 h. The findings of this study are in good agreement with the literature.

**Figure 3:**
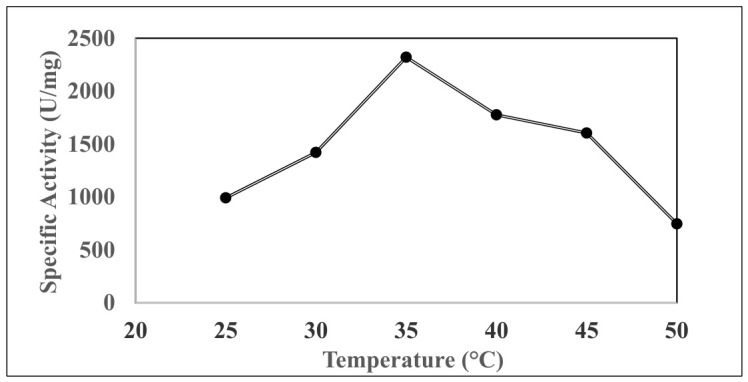
Effect of incubation temperature (20–50 °C) on α-amylase production by *Bacillus simplex* BCHCNZ282B. Enzyme activity is expressed as specific activity (U mg^−1^).

The effect of temperature on maximum enzyme production by the bacterium was investigated. As shown in Figure 3, enzyme production increased within the temperature range of 25–35 °C, whereas a decline in amylase production was observed at higher temperatures. The highest enzyme production was achieved at 35 °C. The reduced enzyme synthesis at temperatures higher than 35 °C could be due to thermal shock on enzyme synthesis in cells. From these findings, *B. simplex* BCHCNZ282B is capable of mesophilic growth while retaining high levels of α-amylase biosynthesis at moderate temperatures. Hmidet et al. [22] reported that maximum amylase production by *Bacillus licheniformis* NH1 occurred at 37 °C.

**Figure 4:**
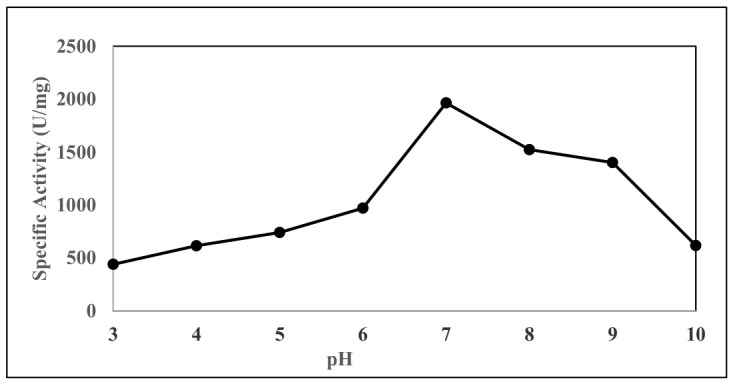
Effect of initial medium pH (3.0–10.0) on α-amylase production by *Bacillus simplex* BCHCNZ282B. Enzyme activity is expressed as specific activity (U mg^−1^).

pH is one of the key parameters affecting enzyme production. The results showed that enzyme production increased from pH 3 onward, reached a maximum at pH 7, and decreased at higher pH values (Figure 4). The decrease in enzyme production with increased acidity and alkalinity may be attributed to poor nutrient uptake, inhibited cell growth, and changes in the gene expression of enzymes responsible for α-amylase synthesis. This suggests that neutral pH is best suited to produce enzymes by *B. simplex* BCHCNZ282B. In the literature, the studies by Hmidet et al. [22], Asgher et al. [23], and Saxena et al. [24] have reported that maximum α-amylase production is reached under neutral-pH conditions.

### 3.3. Effects of Different Carbon and Nitrogen Sources on α-Amylase Production

**Figure 5:**
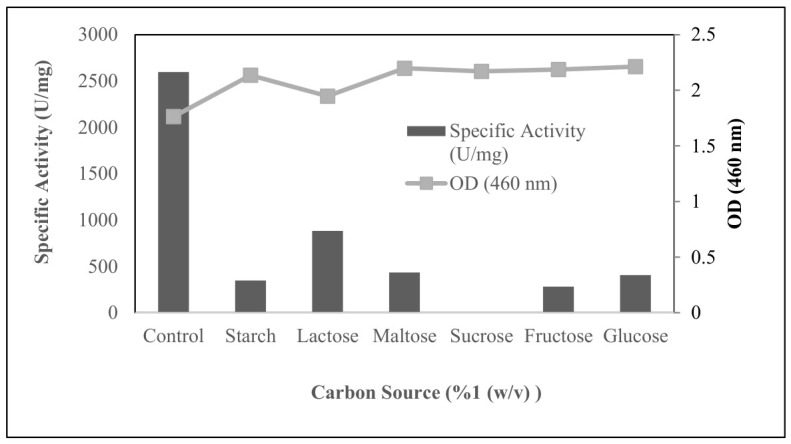
Effects of different carbon sources on α-amylase production by *Bacillus simplex* BCHCNZ282B under optimized cultivation conditions.

The effects of carbon and nitrogen on amylase production from bacteria produced under optimal conditions were examined. According to Figure 5, unlike starch and glucose, sucrose supported relatively low α-amylase production, indicating reduced induction of enzyme synthesis rather than inhibition of enzyme activity. Regarding bacterial growth, all carbon sources supported higher growth than the control.

This study examined the effects of different incubation parameters and nutrient sources on α-amylase production by *B. simplex* BCHCNZ282B. The results revealed that carbon and nitrogen sources significantly influenced enzyme production, with starch, maltose, and fructose promoting α-amylase production, while sucrose exhibited a strong inhibitory effect. These findings agree with those of Agüloğlu Fincan and Enez [25], who reported that sucrose, glucose, and lactose completely suppressed α-amylase activity in *Geobacillus stearothermophilus*, while maltose maintained activity levels close to the control. Similarly, Ashger et al. [23] and Saxena et al. [24] observed that glucose and sucrose inhibited amylase biosynthesis in various *Bacillus* strains, indicating a general pattern of catabolite repression caused by readily metabolizable sugars. The effectiveness of starch as a carbon source observed in our study is further supported by Al-Kabir et al. [26], who demonstrated that maximum α-amylase production by *Bacillus* sp. was achieved when 2% starch was incorporated into the culture medium. Likewise, Wind et al. [27] found that starch and amylopectin were the most favorable carbon sources for *Bacillus stearothermophilus*, whereas glucose, fructose, and sucrose negatively impacted enzyme yields. Dash et al. [28] also highlighted rice flour (rich in starch) as the most effective carbon source for *Bacillus subtilis* BI19, further emphasizing the utility of complex carbohydrates in promoting enzyme production.

**Figure 6:**
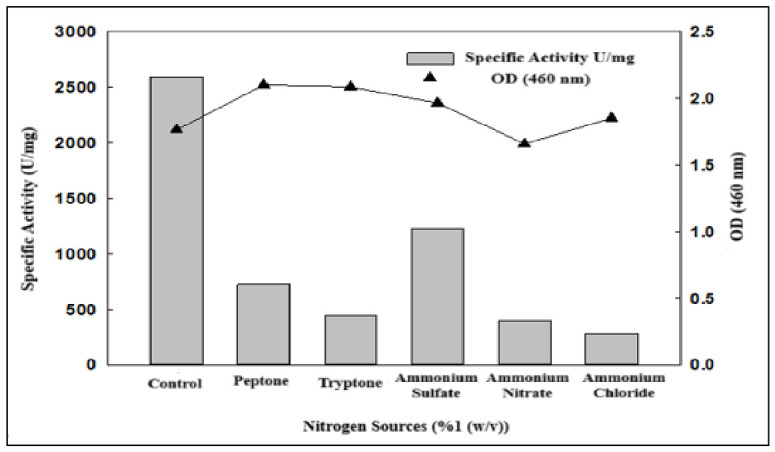
Effects of different nitrogen sources on α-amylase production by *Bacillus simplex* BCHCNZ282B under optimized cultivation conditions.

Regarding nitrogen sources (Figure 6), our results identified ammonium sulfate as lowest inhibitoty effect for α-amylase synthesis, a finding that aligns with Dash et al. [28], who reported its superiority among inorganic nitrogen sources. Although Kumar D. J. et al. [29] proposed sodium nitrate as the most effective nitrogen source for *Bacillus* sp. HPE 10, our study suggests that ammonium salts may offer more consistent enzyme-stimulating effects, at least for *B. simplex* BCHCNZ282B. In contrast, Gangadharan et al. [30] and Mukherjee et al. [31] reported that nitrogen supplementation had no significant impact on amylase production in *B. amyloliquefaciens* and *B. subtilis* DM-03, respectively, highlighting strain-specific metabolic responses. Ortakaya et al. [32] also examined the influence of nutrient sources on α-amylase production by soil-isolated *Bacillus* sp., noting that nitrogen sources generally led to enzyme activity levels comparable to the control, while all tested carbon sources resulted in lower yields. This contrasts with our results, in which certain carbon sources, especially starch and maltose, positively influenced enzyme synthesis, suggesting that *B. simplex* BCHCNZ282B may have more efficient carbohydrate metabolism pathways or regulatory mechanisms favoring amylase induction. These findings underscore the critical role of culture medium composition in microbial enzyme production. The data strongly suggest that polysaccharides such as starch serve as both energy sources and inducers of amylase gene expression. Furthermore, the observed variability in response to carbon and nitrogen sources across studies emphasizes the necessity for strain-specific optimization to achieve maximum enzyme productivity in industrial fermentation processes.

### 3.4. Effects of Different Culture Media on α-Amylase Production

**Figure 7:**
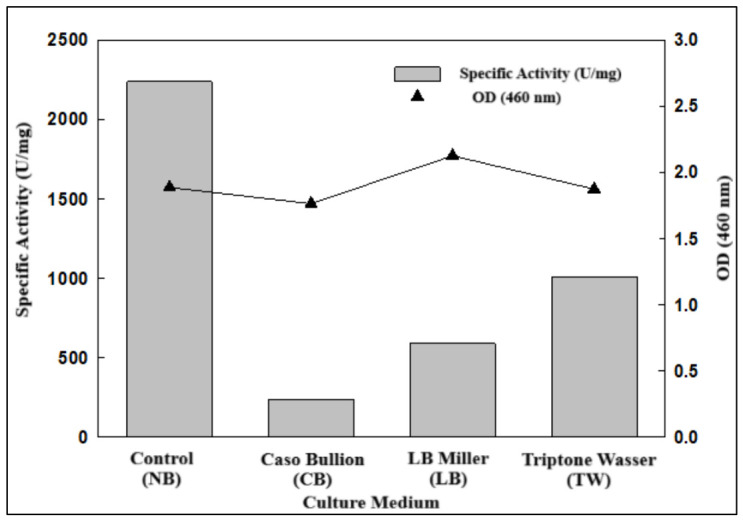
Effects of different culture media (NB, LB Miller, Caso Bullion, and Triptone Wasser) on α-amylase production by *Bacillus simplex* BCHCNZ282B.

In our study, nutrient broth (NB) medium supported the highest specific α-amylase activity by *B. simplex* BCHCNZ282B when compared with other tested media, including LB Miller, Caso Bullion, and Triptone Wasser (Figure 7). In line with our results, Abo-Kamer et al. [33] demonstrated that among five different media tested for α-amylase production by *Bacillus cereus*, the medium containing 10 g/L peptone (Oxoid, Basingstoke, UK), 10 g/L starch, 20 g/L yeast extract, and mineral salts supported the highest specific activity. The high nutrient richness and the presence of complex nitrogen and carbohydrate sources in this medium likely contributed to enhanced enzyme production. Further supporting the importance of starch as a carbon source, Cordeiro et al. [34] identified that the thermophilic *Bacillus* sp. SMA-2 produced maximum α-amylase when cultured in a medium containing soluble starch. Similarly, Smitha et al. [35] and Bukhari et al. [36] reported maximum α-amylase production by *Bacillus thuringiensis* subsp. kurstaki and *Bacillus subtilis* in media containing 2.5% and 1% starch, respectively. These findings reinforce the strong inductive effect of starch on amylase gene expression in *Bacillus* species. Collectively, these results confirm that medium composition is pivotal in optimizing α-amylase production and that starch-based media, whether complex or minimal, consistently support high enzyme activity. The consistency of our findings with previous research strengthens the reliability of NB medium as a cost-effective and efficient medium for industrial amylase production by *B. simplex* BCHCNZ282B.

### 3.5. Purification of α-Amylase from B. simplex BCHCNZ282B

Following the determination of the optimum conditions for α-amylase production by *B. simplex* BCHCNZ282B, the crude enzyme extract was first subjected to 70% ammonium sulfate precipitation, followed by dialysis as an initial concentration and partial purification step. The dialyzed enzyme preparation was subsequently purified by starch-affinity chromatography to further enrich the α-amylase fraction. The purification parameters of the crude extract and the final starch-affinity chromatography step are presented in Table 1. The final starch-affinity chromatography step resulted in a 6.13-fold purification with a recovery yield of 19%. The starch-affinity chromatography step was employed as the final purification step because it provides a relatively simple and cost-effective approach for enriching α-amylase. The purification results obtained in the present study are comparable with those reported previously [19]. Enez [37] reported that α-amylase purified from Bacillus sp. exhibited a 29.8-fold purification with a recovery yield of 9.2%. Ölmez [38] obtained a 1.95-fold purification with a recovery of 28.65% for α-amylase from *Bacillus licheniformis* SO7 using starch-affinity chromatography. Similarly, Primarini and Ohta [39] purified α-amylase from Streptomyces sp. using starch adsorption, affinity chromatography, and ion-exchange chromatography, achieving a 126-fold purification with a recovery yield of 2%. Furthermore, Bolton et al. [40] reported a 2.215-fold purification and a recovery yield of 15.4% for α-amylase from *Bacillus flavothermus* using ammonium sulfate precipitation, dialysis, Q-Sepharose ion-exchange chromatography, and Superose 12 gel filtration.

Although the final purification fold obtained in the present study was lower than those reported in some previous studies, the sequential purification strategy combining ammonium sulfate precipitation, dialysis, and starch-affinity chromatography provided an effective and economical purification procedure. The final starch-affinity chromatography step yielded a 6.13-fold purification with a recovery yield of 19%. The reduction in enzyme recovery during purification is consistent with the losses commonly observed during precipitation, dialysis, and chromatographic separation.

### 3.6. Thermal and pH Stability of Purified α-Amylase from B. simplex BCHCNZ282B

**Figure 8:**
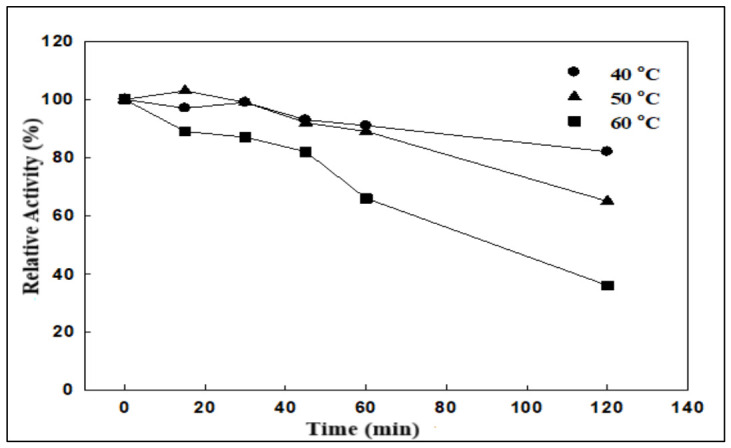
Thermal stability profile of purified α-amylase from *Bacillus simplex* BCHCNZ282B after incubation at 40, 50, and 60 °C. Residual enzyme activity (%) was determined under standard assay conditions.

The stability of an enzyme at various temperatures and pHs is a vital consideration when selecting enzymes for industrial purposes. Thus, the pH and temperature stability of the purified α-amylase enzyme in this study was investigated. The enzyme retained 91% and 89% of its activity after 60 min at 40 and 50 °C, respectively. At 60 °C, the enzyme maintained 82% of its initial activity for up to 45 min (Figure 8). The high residual activity after prolonged incubation indicated the enzyme’s ability to withstand higher temperatures, which ensures efficient enzyme function during industrial processes that require higher temperatures.

**Figure 9:**
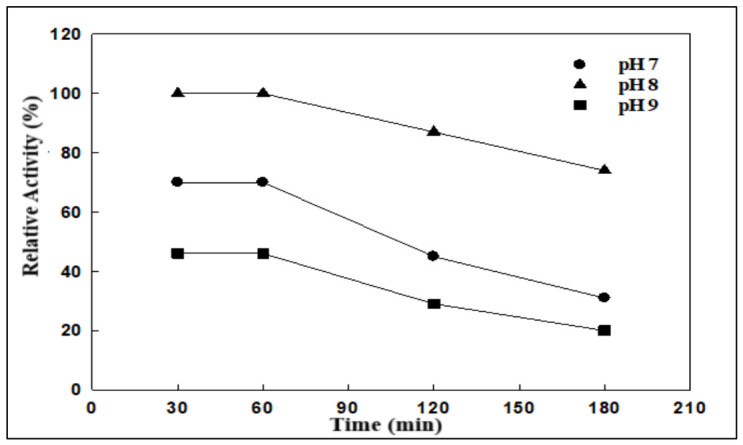
pH stability profile of purified α-amylase from *Bacillus simplex* BCHCNZ282B following preincubation for 60 min in different buffer systems. Residual enzyme activity (%) was determined under standard assay conditions.

Additionally, the enzyme preserved 100% of its activity at pH 8.0 after 60 min of incubation, indicating strong pH stability under alkaline conditions (Figure 9). The enzyme’s stability at an alkaline pH indicates its potential usefulness in detergents and starch processing given the alkalinity of these environments. Although the maximum growth temperature for the isolated *B. simplex* BCHCNZ282B was 37 °C, characteristic of a mesophilic organism, the optimal temperature for α-amylase activity was 50 °C. This finding aligns with the growing body of literature showing that mesophilic microorganisms can produce thermotolerant α-amylase. Several mesophilic *Bacillus* species have been reported to secrete thermostable α-amylases. For example, Agüloglu et al. [41] determined that they were stable at 40 °C and 45 °C. Muniasamy et al. [42] found that the α-amylase purified from *Bacillus simplex* ON754233 was thermostable at 60 °C and remained stable under alkaline conditions (pH 8.0). In addition, Rakaz et al. [43] reported that the enzymes purified from *Bacillus cereus* and *Bacillus licheniformis* were both stable at pH 8.0. While both enzymes remained stable at 45 °C for 15 min, the enzyme activity of *B. cereus* was completely lost (100% reduction) after 15 min at 75 °C. The enzyme activity of *B. licheniformis* decreased to 70% under the same conditions.

### 3.7. Effects of Detergents on Activity of Purified α-Amylase

**Figure 10:**
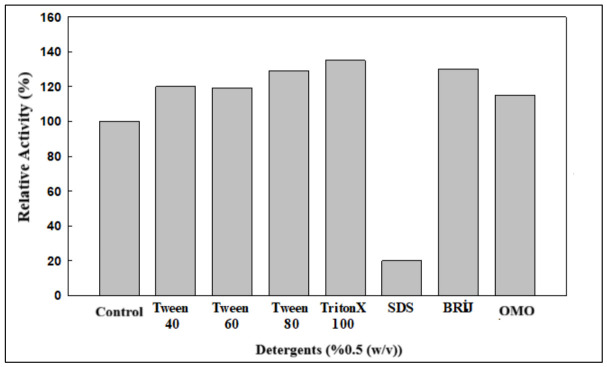
Effects of detergents on activity of purified α-amylase. Residual activities were calculated relative to untreated control enzyme (100%).

The relative activities were calculated by comparing the residual enzyme activity with that of the control. The enzyme exhibited the following relative activities: SDS (20%), Tween-40 (120%), Tween-60 (119%), Tween-80 (129%), Triton X-100 (135%), Brij (130%), and Omo Matik (115%) (Figure 10). These results indicate that the enzyme retained significant activity in the presence of all detergents tested, except SDS, suggesting detergent-tolerant stability. Previous studies have shown that oxidative modifications of amino acids and the presence of disulfide bonds in enzymes can contribute to enhanced thermostability and resistance to oxidative agents [44]. Similarly, this study’s findings suggest that α-amylase exhibits resistance to oxidative agents, further supporting its stability and potential industrial applicability.

### 3.8. Effects of Various Inhibitors on the Activity of Purified α-Amylase

The effects of several inhibitors on the activity of the purified α-amylase were investigated, including the serine-specific inhibitor PMSF, the sulfhydryl group inhibitor 2-mercaptoethanol, the metal chelator EDTA, and organic solvents such as methanol and ethanol (Table 2). Slight increases in relative activity were observed with PMSF, methanol, ethanol, and 2-mercaptoethanol at low concentrations. Such activation effects have been reported for certain microbial α-amylases and may be associated with the stabilization of the enzyme structure, protection against minor oxidative effects, or improved accessibility of the substrate to the catalytic site. In contrast, EDTA significantly reduced enzyme activity, suggesting a possible requirement for metal ions in maintaining catalytic activity and structural stability.

The significant inhibition by EDTA supports the classification of this α-amylase as a metalloenzyme, as reported by Dheeran et al. [45], Du et al. [46], Xie et al. [47], and Zafar et al. [48].

### 3.9. Effect of Metal Ions on Activity of Purified α-Amylase

**Figure 11:**
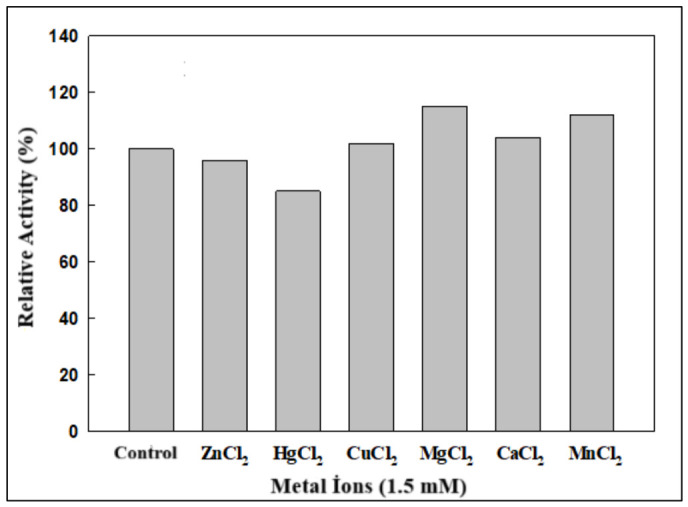
Effects of metal ions on the activity of purified α-amylase. Residual activities were calculated relative to the control reaction without added metal ions.

The impacts of various metal ions, including ZnCl_2_, HgCl_2_, CuCl_2_, MgCl_2_, CaCl_2_, and MnCl_2_, on the activity of the purified α-amylase were assessed by individually adding them to the enzyme assay. The results indicated that the addition of MgCl_2_ (115%), MnCl_2_ (112%), CuCl_2_ (102%), and CaCl_2_ (104%) enhanced enzyme activity, whereas ZnCl_2_ (96%) and HgCl_2_ (84%) caused partial inhibition (Figure 11). These findings are consistent with previous reports indicating that Mg^2+^ and Mn^2+^ may exert stimulatory effects on bacterial α-amylases [49]. In this study, Mg^2+^ and Mn^2+^ produced moderate increases in enzyme activity, whereas Ca^2+^ and Cu^2+^ caused slight enhancements. Most tested metal ions did not markedly inhibit enzyme activity, although Hg^2+^ showed a more pronounced inhibitory effect. Rajesh et al. [50] similarly reported that α-amylase activity purified from *Bacillus* sp. PM06 was enhanced in the presence of Mg^2+^, Co^2+^, Ca^2+^, and Mn^2+^ ions, while Cu^2+^ and Ni^2+^ ions exerted inhibitory effects. In another study, Asoodeh et al. [51] found that α-amylase activity from *Bacillus* sp. DR90 was stimulated by Ba^2+^, Fe^2+^, and Mg^2+^ ions, whereas Hg^2+^ and Zn^2+^ ions reduced enzyme activity.

### 3.10. Purification and Molecular Mass Determination of α-Amylase

**Figure 12:**
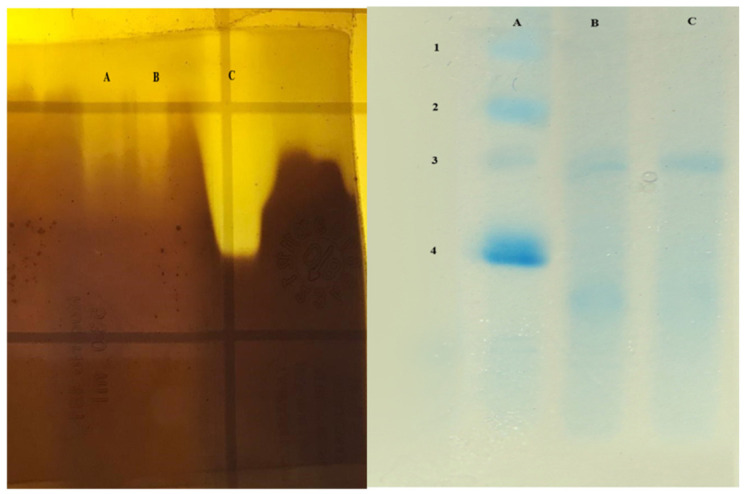
(**Left panel**)—starch gel: (A) dialysis, (B) starch-affinity chromatography, and (C) commercial amylase; (**Right panel**)—SDS-PAGE gel: (A) standard protein mix (1.116 kDa, 2.97 kDa, 3.72 kDa, and 4.29 kDa), (B) dialysis, and (C) starch-affinity chromatography.

Enzymatic purification of α-Amylase from Bacillus simplex BCHCNZ282B was performed using starch-affinity chromatography. According to the results obtained by analyzing the purified fraction through SDS-PAGE electrophoresis (Lane C), the purified fraction contained one distinct protein band with the molecular mass of about 70 kDa (Figure 12). This band was identified as α-amylase since it was found in the fraction with enzymatic activity obtained during the process of purification, and its molecular mass corresponded to that of other bacterial α-amylases. Starch activity gel additionally proved the presence of amylolytic activity in the fractions purified by the dialysis and starch-affinity chromatography. The same amount of total protein was loaded in each of the lanes in order to compare enzyme activity. There was an observed starch hydrolysis zone in the starch-affinity chromatography fraction, which proved the presence of amylolytic activity in the purified 70 kDa protein. Commercial α-amylase was used as a positive controlMolecular mass estimation at about 70 kDa is consistent with those observed in other bacterial α-amylases. The molecular mass of 74 kDa was observed in the case of α-amylase isolated from Bacillus licheniformis SO-B3 [20], whereas the molecular mass of 73 kDa was found in α-amylase isolated from Bacillus mojavensis [52]. Thus, both the SDS-PAGE pattern and the activity staining results confirm that the protein having molecular mass around 70 kDa is indeed the purified α-amylase isolated from B. simplex BCHCNZ282B.

## 4. Conclusions

This study explored the optimization of the culture conditions for α-amylase production by *B. simplex* BCHCNZ282B and the purification and characterization of the enzyme. The enzyme was isolated using starch-affinity chromatography in one step, leading to an approximately six-fold increase in the enzyme’s specific activity. The isolated enzyme showed good biochemical characteristics, such as stability at wider temperature ranges and pH values, as well as stability against various non-ionic detergents. The enzyme’s sensitivity to SDS, however, implies that its compatibility depends on the kind of detergent used. This demonstrates the enzyme’s potential for application in biotechnology and industry. However, more research must be conducted for this purpose.

## Figures and Tables

**Table 1:** α-amylase purification steps.

Purification Steps	Total Protein (mg)	Total Activity (U)	Specific Activity (U/mg)	Purification Fold	Yiel (%)
Crude extract	11.505	23,841	2072	1	100
Starch-Affinity Method	0.365	4651	12,716	6	19

**Table 2:** The effects of inhibitors on pure enzyme.

	1 mM	5 mM	10 mM
Control	100	100	100
PMSF	115	113	39
Methanol	118	111	98
2-Mercaptoethanol	111	100	84
EDTA	94	21	16
Ethanol	118	113	111

## Data Availability

The raw data supporting the conclusions of this article will be made available by the authors on request.
